# Investigating the Deformation Characteristics of Buried High-Density Polyethylene Pipes: Considering the Effect of Sequentially Applying Pressure and Elevating Temperature

**DOI:** 10.3390/polym14183779

**Published:** 2022-09-09

**Authors:** Yuchen Zhang, Jianyong Shi, Zhanlei Liu, Zhenming Sun, Xun Wu

**Affiliations:** 1 Key Laboratory of Ministry of Education for Geomechanics and Embankment Engineering, Hohai University, Nanjing 210024, China; 2 College of Mechanics and Materials, Hohai University, Nanjing 210024, China

**Keywords:** deformation characteristics, strain, high-density polyethylene pipe, landfill, elevated temperature, pipe profile

## Abstract

High-density polyethylene (HDPE) materials have many applications in the municipal solid waste (MSW) landfills. HDPE gravity drainage pipes are commonly utilized in MSW landfills because of the polymer’s resistance to harsh chemical conditions. When landfill wastes are freshly filled, the weight acting on the leachate collection pipe increases. The temperature of the leachate collection pipe increases as a result of the heat produced by the decomposition of organic components after waste filling. In this paper, the effects of sequentially applying pressure and elevating temperature on the deformation characteristics (such as deformations and strains) of HDPE pipes are investigated. Measurements of pipe deformations and circumferential strains from model experiments in which 110 mm HDPE pipes were backfilled with sand and subjected to 300 kPa of maximum vertical pressure at temperatures of 20, 60 and 80 °C showed the following results: (1) a classification of pipe behavior relative to the surrounding soil stiffness is advantageous for HDPE pipe design; (2) when temperature increases to 60 °C and 80 °C, the strain distribution around the pipe changes from V-shaped to U-shaped, and the pipe deformation profile changes from elliptical to rectangular; (3) when temperature increases from 20 °C to 60 °C, the vertical and horizontal pipe deflections increase by a factor of 1.08~1.19; (4) when temperature increases from 60 °C to 80 °C, the vertical and horizontal pipe deflections increase by a factor of 1.15~1.31; and (5) the existing analytical method that considers two extreme interfaces can capture the deformations measured in the model test well. In addition, preliminary recommendations for the design of leachate collection pipes are provided based on the analysis of differences in pipe profile versus temperature.

## 1. Introduction

High-density polyethylene (HDPE) pipes are an essential component of the leachate collection system of a number of municipal solid waste (MSW) landfills [1,2,3,4,5,6,7,8]. This is mostly because high-density polyethylene is a polymer that is resistant to the harsh chemical conditions found in MSW landfills. The large earth pressures and elevated temperatures in landfills can cause HDPE pipes to collapse, resulting in clogged drainage systems and a rise in leachate levels [8]. To ensure the economic and safe operation of leachate collection pipes in these harsh environments, it is vital to understand the deformation properties of buried HDPE pipes [8].

Existing research quantifies the effect of coarse gravel and backfill construction on landfill leachate collection pipe deformation [9,10]. Krushelnitzky and Brachman [6] investigated the deformation of buried HDPE pipes with backfill materials of various particle sizes and densities under deep burial scenarios using laboratory testing. Khatri et al. [11] utilized a parallel plate test in the laboratory to assess the stress and deformation of HDPE pipes reinforced with steel. Through model studies, Zhou et al. [12] evaluated the peak displacement and total displacement of HDPE pipes during backfilling. Li et al. [13] investigated the mechanical behavior of polyethylene pipes under strike-slip fault movements. In most of these studies, temperature variations were not considered.

Recent studies indicate, however, that municipal solid waste dumps may be far hotter than anticipated a decade ago. Presently, the liner temperature of a conventional municipal solid waste landfill is anticipated to be between 30 and 40 °C, whereas the liner temperature of a municipal solid waste dump may reach 60 to 80 °C [14]. Krushelnitzky and Brachman [8] investigated the deformation properties of underground HDPE pipes at various temperatures (22 °C, 50 °C and 80 °C) and pressures (200 kPa, 400 kPa, and 500 kPa). In Krushelnitzky and Brachman [8], the interior space of the buried HDPE pipe was heated by an air heat pump at the beginning of the experiment. After the HDPE pipe reached the required temperature, vertical pressure was applied. Newly filled waste in a landfill increases the load operating on leachate collection pipes, and the decomposition of organic components following waste filling contributes to an increase in waste temperature. Moore [3] described the response of a buried HDPE pipe as a complex soil-structure interaction problem, with the surrounding soil providing load and support for the pipe. It is often assumed that the elastic modulus of polyethylene falls as its temperature rises [15,16]. To accurately replicate the ambient environment of leachate collection pipes, it is essential to analyze the deformation characteristics of buried HDPE pipes subject to the same processes that occur in landfills.

In previous studies, strain gauge measurements at ambient temperatures were used to forecast the deformation profiles of buried flexible pipes [17,18], while Krushelnitzky and Brachman [8] did not report the deflected form of buried HDPE pipes under increased temperatures. In addition to pipeline deformation and deformation profile shapes, the deformation characteristics of an HDPE pipeline buried in a landfill should be investigated.

This work aims to evaluate the effect of successively applying pressure and elevating temperature on the deformation characteristics of a buried HDPE pipe. Large-scale physical tests were conducted to assess the deformation and circumferential strain of HDPE pipes backfilled with sand at 20 °C, 60 °C and 80 °C and at up to 300 kPa of vertical pressure. The observed pipe deformations and circumferential strains were compared with known analytical solutions and their efficacy was evaluated. In addition, preliminary recommendations for the design of leachate collection pipes are provided based on analyses of differences in pipe profile versus temperature.

## 2. Materials and Methods

### 2.1. Test System and Boundary Conditions

**Figure 1:**
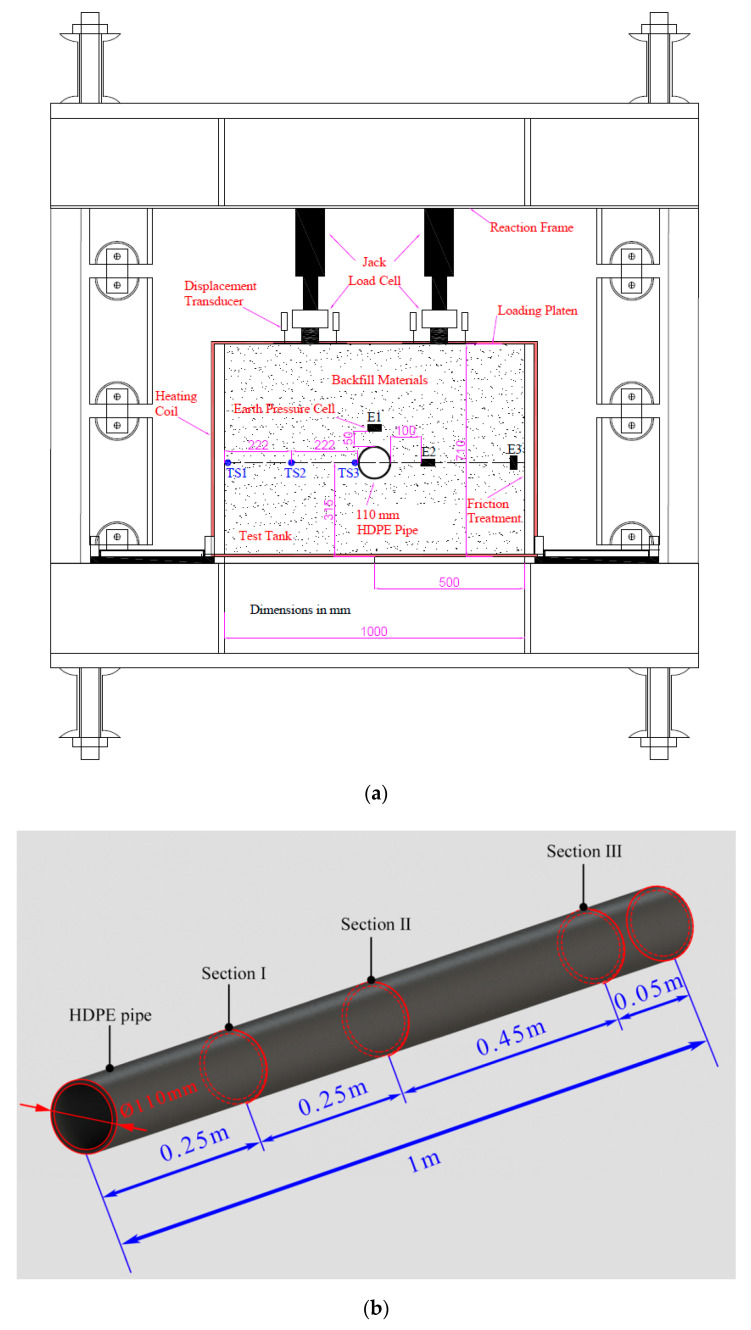

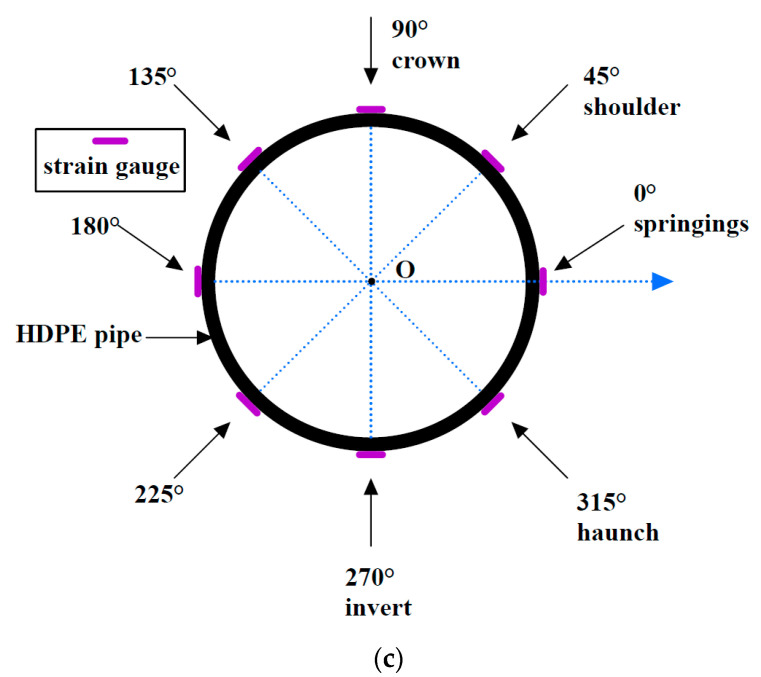
Test system: (**a**) schematic drawing; (**b**) sections for strain gauges arrangement; (**c**) strain gauge distribution on each section.

This study presents the results of tests conducted on specimens using a three-directional mobile loading platform developed by the authors. The platform featured a reaction frame consisting of a self-balancing three-dimensional loading steel structure, two static actuators, an electrohydraulic servo loading control system, and a test tank measuring 1 m in length, 1 m in width, and 0.71 m in height (as depicted in Figure 1).

As shown in Figure 1a, a vertical pressure was applied in the testing tank by the use of an actuator and a loading platen located at the very top of the tank. The walls of the test tank were not allowed to deflect outwards. As a result, it was possible to produce a specimen state in which there was no lateral strain. The walls of the test tank were coated with a double layer of plastic sheeting that had been greased with vaseline so that there would be less friction between the soil and the walls of the tank. According to Krushelnitzky and Brachman [8], the friction angle of the tank wall may be regulated to within 5° with the utilization of the friction treatment method.

To isolate the influence of variations in temperature on the mechanical behaviour of HDPE pipes, electrical heating coils were wrapped around the perimeter of the test tank, including the top and bottom surfaces, to control the temperature at the test tank boundaries.

### 2.2. Pipe

In this investigation, the walls of the evaluated HDPE pipes were flat. Because of the resistance of high-density polyethylene to harsh chemical environments, it is a material that is widely applied in the construction of a variety of municipal and industrial infrastructures. Each sample came from the same batch of materials. The design of a pipe with a plane wall requires the selection of a suitable wall thickness, which is typically represented using the standard dimension ratio (SDR). The standard dimension ratio is defined as the ratio of the pipe’s outer diameter to the pipe’s thickness. The pipes that were examined had an external diameter of 110 mm.

Both the SDR 11 and the SDR 26 pipe were put to the test. The SDR 11 pipe had an average wall thickness of 10 mm, and the SDR 26 pipe had an average wall thickness of 4.2 mm. To an accuracy of within 0.01 mm around the pipe’s center, vertical and horizontal diameter changes of the pipes were measured with displacement transducers. The variations of the inner diameter of the pipes during the tests were also obtain by a laser displacement transducer inside the pipe. There were gaps with a width of 5 mm that were covered with foam boards. These gaps existed between the ends of the pipe and the tank walls. Because of this treatment, the HDPE pipes’ axis deformation was not restricted throughout the testing, and backfills were prevented from entering the interior space of the HDPE pipes. In addition to that, this treatment is effective in preventing the HDPE pipes from becoming jammed up against the tank walls.

According to GB/T13663.2 [19], the material properties of the HDPE pipe used in this paper were tested. The material properties of the HDPE pipe are shown in the Table 1. The HDPE pipes are extrusion-molded. Extrusion-molded PE pipes were investigated in some previous studies [20,21].

The sand was backfilled with sand pourers to the height of the bottom of the pipe. After the HDPE pipe was put on the existing sand, the sand was backfilled with sand pourers to the designed height of the specimen. Sand pouring is a method through which the density and uniformity of backfill soil can be well-controlled [22,23].

During the test, strain gauges were utilized so that the HDPE pipe strain could be determined. Figure 1b,c present an illustration of the configuration of the strain gauges. The strain gauges were placed at 0°, 45°, 90°,135°, 180°, 225°, 270° and 315° in Section I, II, and III of the HDPE pipe.

### 2.3. Backfill Materials

Before testing, river sand was used to fill the test tank, and then it was allowed to dry before being screened using mesh sizes of 2.36 mm. Sand pourers were used to prepare the specimens. The characteristics of the materials used for backfilling are detailed in Table 2.

Poorly graded and medium-coarse consistency sand was used in this study, as specified by [24].

During the experiments, measurements were taken of the earth pressure distributions in the backfill materials. During the testing, a strain-type micro earth pressure cell with an accuracy of 0.25 percent FS was utilized. The range of the cell was from 6 to 600 kPa. The diameter of the earth pressure cell was 35 mm, while its thickness measured 15 mm. The earth pressure cell had a temperature range of operation that went from −40 °C to 125 °C.

### 2.4. Control of Vertical Pressure and Temperature during the Test

The procedure for the tests conducted in this study was designed to simulate the sequential application of pressure and elevated temperature on leachate collection pipes in MSW landfills.

With each additional layer of waste that is placed in a landfill, there is a corresponding increase in the amount of pressure exerted on the HDPE pipe that is used for leachate collection. The height of the daily filled waste is assumed to be 5 m [25], and the average density of waste is assumed to be 10 kN/m^3^ in this study [26,27]. As a result, the load increment that is operating on the buried HDPE pipe is assumed to be 50 kPa throughout this study. The loading rate of the vertical pressure was determined to be 50 kPa per 40 min. This rate was also utilized by Krushelnitzky and Brachman [6,8]. During the course of the test, the maximum vertical pressure was 300 kPa.

Following completion of the waste filling process, heat generation occurs in landfills as a result of the degradation of organic components. This heat generation correlates to an increase in the temperature of the waste. According to Yeșiller et al. [14], the temperatures of the waste in a landfill range anywhere from 20 °C. to 80 °C. As a result, following the application of the maximum vertical pressure, the surrounding temperature was regulated to 20, 60 and 80 °C.

In this particular piece of research, temperature sensors with an accuracy of 0.2 °C. and a temperature range of −50 °C to 500 °C were utilized so that the temperature of the HDPE pipe could be brought up to the desired level. As can be seen in Figure 1, the temperature sensor designated TS1 is positioned so that it is in close proximity to the side wall of the test tank, the temperature sensor designated TS3 is positioned so that it is in close proximity to the HDPE pipe, and the temperature sensor designated TS2 is positioned so that it is in close proximity to both TS1 and TS3.

The details of the tests conducted in this study are outlined in Table 3.

## 3. Results

Even though many readings were collected over the course of the test procedure, the ones discussed are limited to those that pertain to the strain and deformation of the pipe walls. Additional information can be found in [28]. Measurements of circumferential strain were taken at intervals of 45° around the pipe’s outer surface. These data have been plotted in the strain profile either linearly or circularly for ease of evaluation. This work follows a sign convention in which tensile strains are positive and compressive strains are negative. This convention is used throughout the study.

### 3.1. Circumferential Strain at 20 °C

The circumferential strain of each section after the application of vertical pressure to an HDPE pipe at a temperature of 20 °C is shown below.

**Figure 2:**
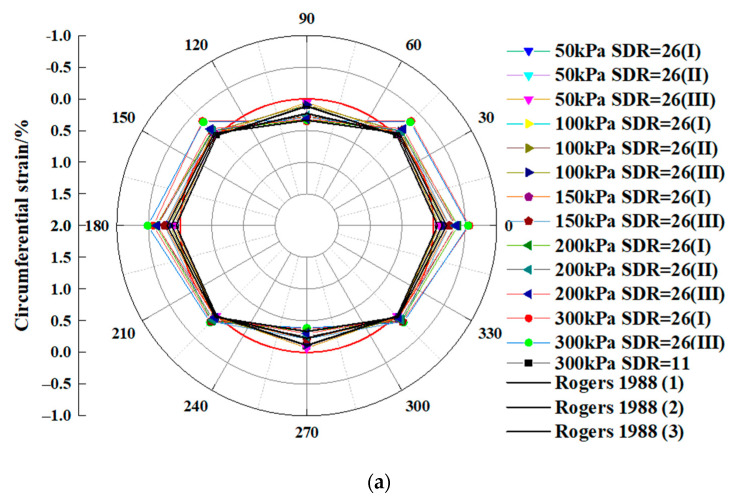

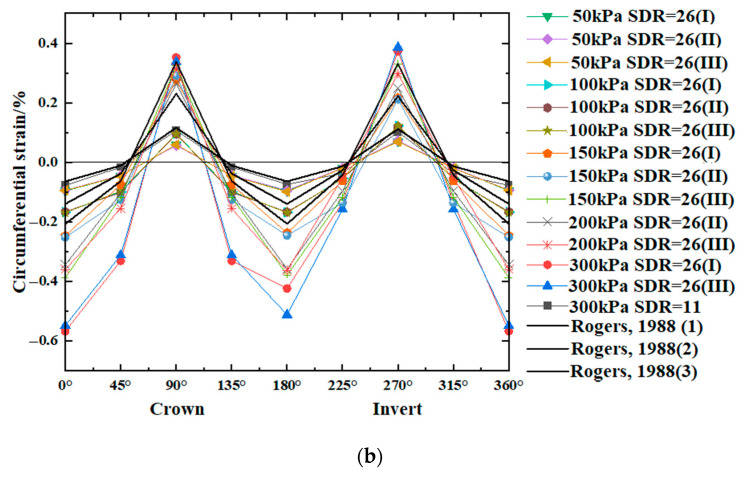
Strain profiles for the load test: (**a**) circular axis; (**b**) linear axis.

Figure 2 depicts the amount of strain in Sections I, II, and III of an HDPE pipe when a vertical pressure was applied. The ambient temperature was 20 °C. In each test, the rise in the vertical pressure caused an increase in the circumferential strain placed on the HDPE pipe. Positive strains, indicating tensile strain, were measured at 90° and 270°. The stresses at 0°, 45°, 135°,180°, 225° and 315° have negative values, which indicated that they were compressive strains. The stresses measured at angles of 90° and 270° represented the highest possible tensile strain at the pipe crown and invert. The values of the circumferential strain at 0° and 180° had the same sign and were comparable, which was compatible with the symmetric locations of 0° and 180°. Both the stress state and the position of the two points were symmetrical. Symmetry existed between the two points. While the strain at an angle of 90° was positive, indicating tensile strain, the strain at an angle of 0° was negative, indicating compressive strain. There was a transition zone from compression to tension that existed between 0° and 90°. Analogously, the region between 90° and 180° constituted a transition zone from tension to compression.

As shown in Figure 2, the findings of this study were compared to those of Rogers [29]. Rogers [29] conducted experiments in which underground flexible pipes were subjected to an externally imposed vertical pressure at room temperature. At a temperature of approximately 20 °C, the test conditions described in Rogers [29] were comparable to those described in this study. As shown in Figure 2, the findings of this study at 20 °C exhibited variances that were comparable to those found in [29], indicating that the findings at 20 °C were credible.

The strain profiles that were taken from the pipe walls during this test are displayed on a circular axis in Figure 2a, from which it is evident that the strain profiles have an elliptical shape. When the same data were averaged and shown on a linear axis (Figure 2b), it was possible to have a better understanding of the relative magnitudes of strain. Each curve followed the same general pattern, which consisted of a high tensile strain in the pipe crown, with equal and opposite strains at the springing (compressive). The curve for river sand showed a very minor departure from the V-shaped pattern. The pipe crown and invert stresses were the highest, while the strain at the haunches was the lowest.

In addition, the strain profiles allowed for the prediction of the shape that the pipe would have once it was distorted. Howard [18] demonstrated that elliptical deformation is associated with a V-shaped strain profile. This is because the resistance to the movement of the side of the pipe causes the elliptical deformation to be associated with a V-shaped strain profile. It is thus possible to conclude, based on these data, that the deformation of the pipes at the end of the tests was nearly elliptical. The pipe that was placed in river sand showed a propensity to flatten at the crown and swell somewhat at the shoulders. It is important to mention that the amount of pipe deformation associated with these profiles occurred at a temperature of approximately 20 °C.

### 3.2. Circumferential Strain at 60 °C and 80 °C

**Figure 3:**
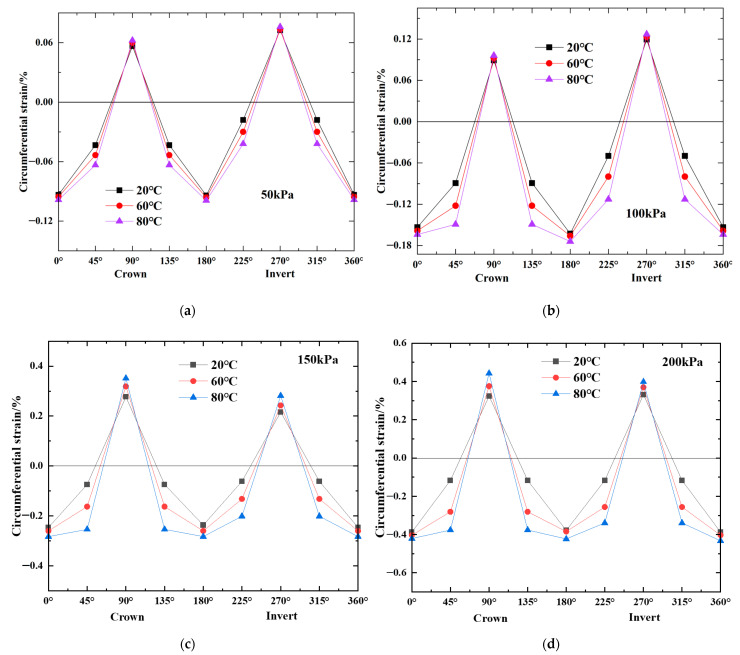
Strain profiles for the load test versus temperature: (**a**) 50 kPa vertical pressure; (**b**) 100 kPa vertical pressure; (**c**) 150 kPa vertical pressure; (**d**) 200 kPa vertical pressure.

**Figure 4:**
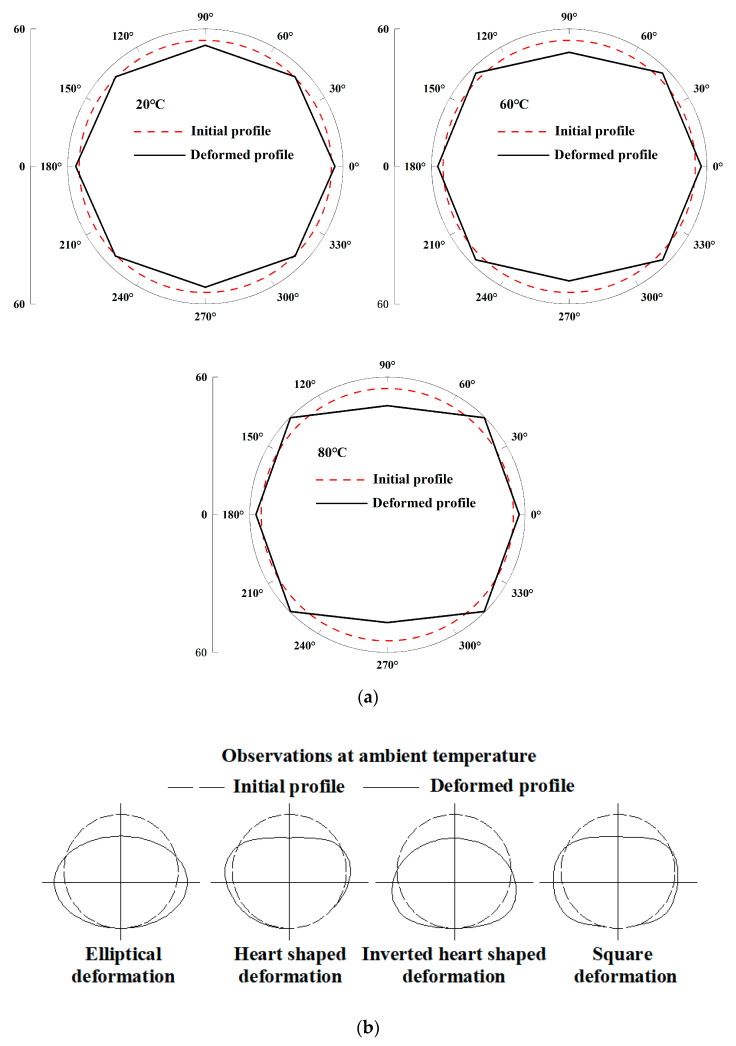
The pipe deformation profile: (**a**) measured in this paper (200 kPa); (**b**) types of deformation reported in Howard [17] at ambient temperature.

As shown in Figure 3, when the temperature was increased to 60 °C and 80 °C, the strain at 0°, 90°, 180° and 270° increased slightly. On the other hand, the strain at 45°, 135°, 225° and 315° increased significantly. As a direct consequence, the strain distribution throughout the three portions changed from a V shape to a U shape. The highest strain around the pipe was found anywhere between 0° and 270° when the strain distribution was in the form of a V. However, when the distribution of the strain changed into a U-shape, the maxima at 45°, 135°, 225° and 315° showed the maximum strain. In addition, as shown in Figure 4a, the ellipse-to-rectangle transition in the pipe deformation profile occurred when the strain distribution changed from a V-shape to a U-shape. The noncontact approach that was used to obtain the full shape of the HDPE pipe was found to be rather accurate, in contrast with the photographic technology that was published in earlier literature [17]. The U-shaped deflection that was discussed before is analogous to what Howard [17] meant to describe when he discussed rectangular deflection. In this regard, rectangular is perhaps a more accurate description because the pipe experiences flattening at the crown and invert, with few changes in curvature (i.e., low strain) at the springing. This flattening occurs at the crown and invert of the pipe. According to Howard [17], a distortion in the shape of a square (Figure 4b) occurred after the backfill that surrounded the pipe was well compacted. Tests in Howard [17] were conducted at ambient temperature, and different types of pipe deformation profile (Figure 4b) were observed with different backfill conditions during the tests. In the prior body of research, it was speculated that the qualities of the pipe would remain constant over time. However, the qualities of the pipe are subject to changes in landfills due to the fluctuating temperature of the waste. It is a widely held belief that temperature has an effect on the mechanical characteristics of polyethylene, specifically, high temperature reduces the material’s elastic modulus [15,16]. Not only can the compactness of the backfill affect the shape of the pipe’s deformation, but the shape of the pipe’s deformation can also be affected by the relative stiffness of the pipe to the backfill.

The design for the currently used landfill leachate collection pipes is primarily based on a widely used calculation method for buried pipe design. This method is known as the Iowa formula [30,31,32,33]. In the Iowa formula, the deformation of the buried HDPE pipe is assumed due to the stability of the elliptical shape. The maximum vertical and horizontal deformations have the greatest impact on the design of the HDPE pipe model. The Iowa calculation does not take into consideration the consequences that may be caused by the increased temperature that occurs in landfills. The findings presented in this study demonstrate that the high temperatures prevalent in landfills lead to deformations in HDPE pipes that result in nonelliptical pipe shapes. Therefore, while designing a pipe that collects leachate from a landfill, in addition to taking into consideration the maximum vertical and horizontal deformations, one should also take into consideration the stress and strain to determine whether the pipe is stable.

### 3.3. Creep Measurements of the HDPE Pipe

**Figure 5:**
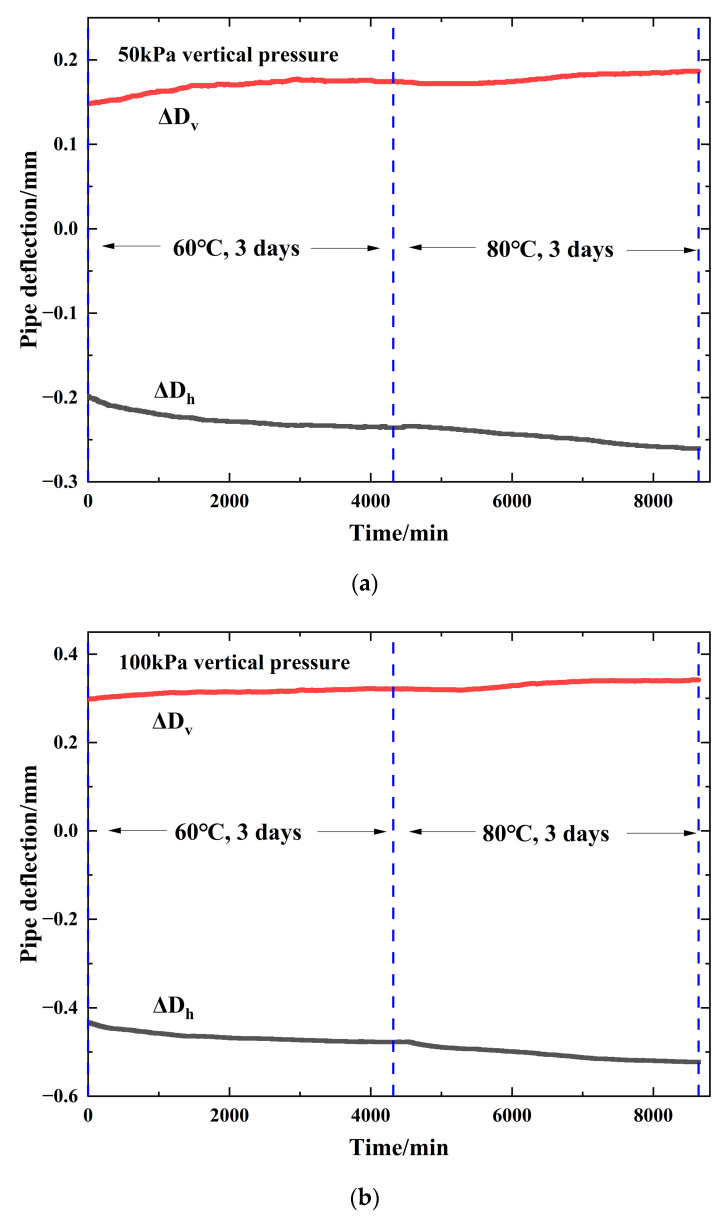

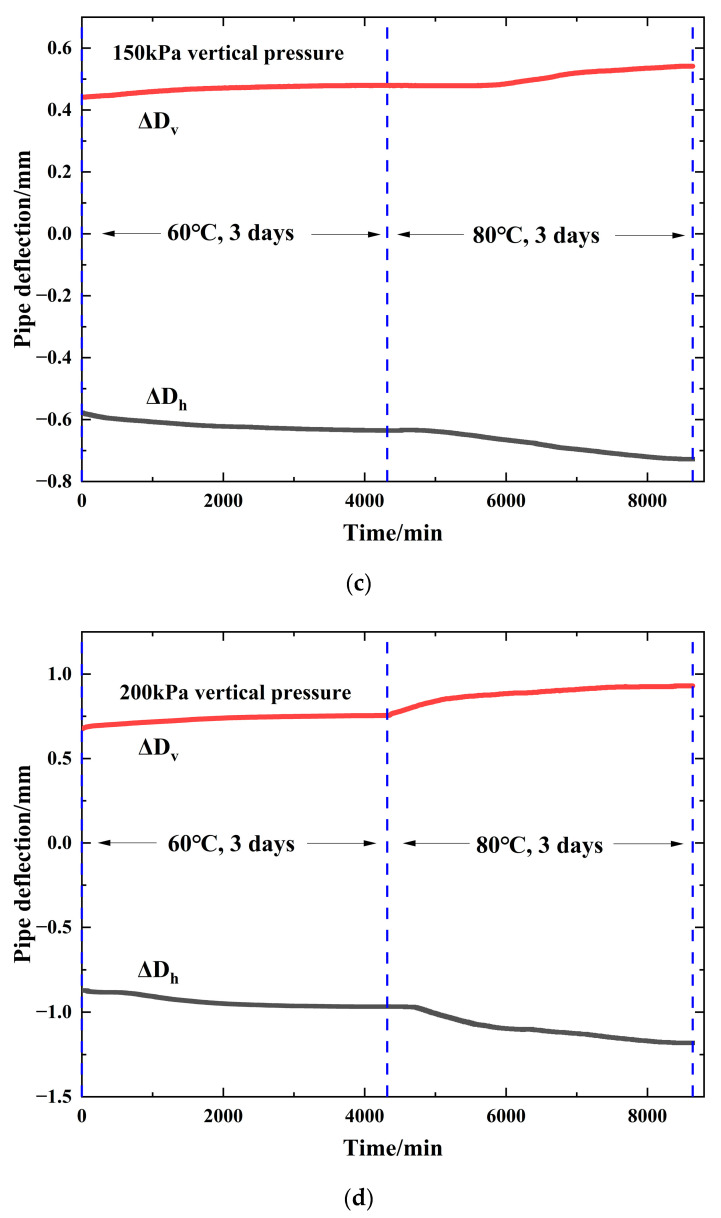
The creep measurements of the HDPE pipe in the duration of the test: (**a**) 50 kPa vertical pressure; (**b**) 100 kPa vertical pressure; (**c**) 150 kPa vertical pressure; (**d**) 200 kPa vertical pressure.

The long-term mechanical behavior of buried pipe is very important for pipe design [34], and the creep measurements of the HDPE pipe were obtained during the tests in this paper. The creep measurements of the HDPE pipe in the duration of the test are shown in Figure 5. The vertical deformation (ΔD_v_) is negative, indicating compression. The horizontal deformation (ΔD_h_) is positive, indicating tension.

As shown in Figure 5a, when the vertical pressure was applied to 50 kPa at the beginning of the test (the ambient temperature was 20 °C), the ΔD_v_ of the HDPE pipe was −0.1983 mm and the ΔD_h_ of the HDPE pipe was 0.1482 mm. After the temperature of the specimen was increased to 60 °C for three days, the ΔD_v_ of the HDPE pipe increased to −0.2357 mm, which was increased by a factor of 1.19, and the ΔD_h_ of the HDPE pipe increased to 0.1750 mm, which was increased by a factor of 1.18. After the temperature of the specimen was increased to 80 °C for three days, the ΔD_v_ of the HDPE pipe increased to −0.2604 mm, which was increased by a factor of 1.31, and the ΔD_h_ of the HDPE pipe increased to 0.1865 mm, which was increased by a factor of 1.26.

The creep measurements of the HDPE pipe in the duration of the test under 50 kPa, 100 kPa, 150 kPa, and 200 kPa vertical pressures are quantified and shown in Table 4. As shown in Table 4, when temperature increases from 20 °C to 60 °C, the vertical and horizontal pipe deflections increase by a factor of 1.08~1.19, and when temperature increases from 60 °C to 80 °C, the vertical and horizontal pipe deflections increase by a factor of 1.15~1.31.

## 4. Discussion

### 4.1. Comparison with Analytical Analysis

Calculations of pipe deflections were performed at temperatures of 20 °C, 60 °C and 80 °C using an analytical approach provided by Zhang et al. [35] based on the elastic soil-structure interaction solution. In Zhang et al. [35], the soil and the pipe are both modelled as idealized versions of linear elastic materials, with properties such as elastic modulus E and Poisson’s ratio v. The elastic modulus of the soil was calculated to be 40 MPa, and Poisson’s ratio was calculated to be 0.18. These were chosen after values obtained in triaxial compression tests at acceptable stress levels were analyzed, and then those values were used to make selections. At temperatures of 20 °C, 60 °C and 80 °C, the elastic moduli for the pipe were calculated to be 343, 195, and 105 MPa, respectively. The elastic modulus at 20 °C was supplied by the provider for the pipe, and the elastic moduli at 60 °C and 80 °C were extrapolated based on Zhang et al. [35], as expressed in Equation (1):

(1) $$\frac{E_{c}}{E_{20℃}}=1.4787e^{-0.017T}$$

where *E_c_* = the elastic modulus of the HDPE pipe at certain temperature (kPa); *E_20_*_°C_ = the elastic modulus of the HDPE pipe at 20 °C (kPa); *T* = the temperature of the HDPE pipe (°C).

According to Zhang et al. [35], the deformations of the pipe at *θ* = 45° were determined by Equation (2):

(2) $$\begin{array}{c} u_{r}=\frac{1-v_{c}{}^{2}}{E_{c}}\{-a_{0}r^{-1}+2b_{0}r+(-2a_{2}r+2a_{2}^{1}r^{-3}+4b_{2}^{1}r^{-1})\cos2\theta -\frac{1}{2}\frac{E_{c}}{1-v}\alpha \cdot \Delta Tr\\ -\frac{v_{c}}{1-v_{c}}[a_{0}r^{-1}+2b_{0}r+(2a_{2}r+4b_{2}r^{3}-2a_{2}^{1}r^{-3})\cos2\theta -\frac{1}{2}\frac{E_{c}}{1-v}\alpha \cdot \Delta Tr]\}+(1+v)\alpha \cdot \Delta Tr \end{array}$$

where *u_r_* = radial deformation of the HDPE pipe (mm); *r* = radius to the centroid of the pipe (m); *E_c_* = the elastic modulus of the HDPE pipe (kPa); *v_c_* = Poisson’s ratio of the HDPE pipe; *a_x_* and *b_x_* are undetermined coefficients depending on the boundary conditions.

**Figure 6:**
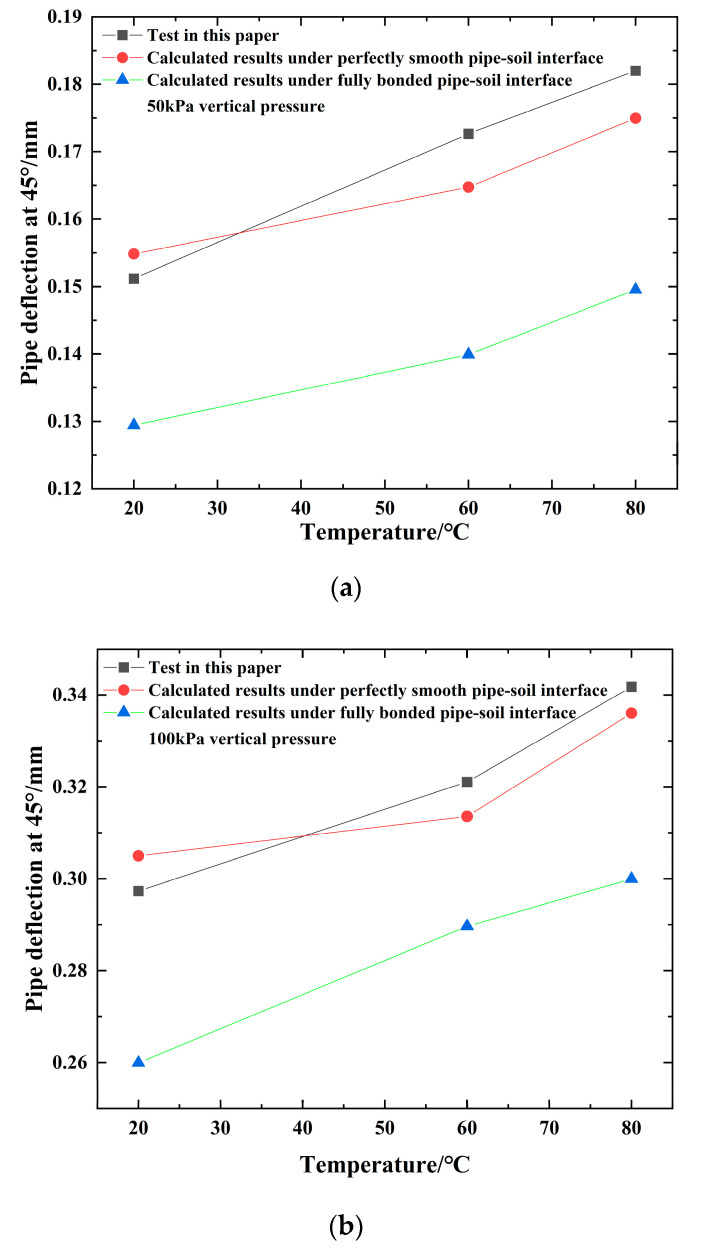

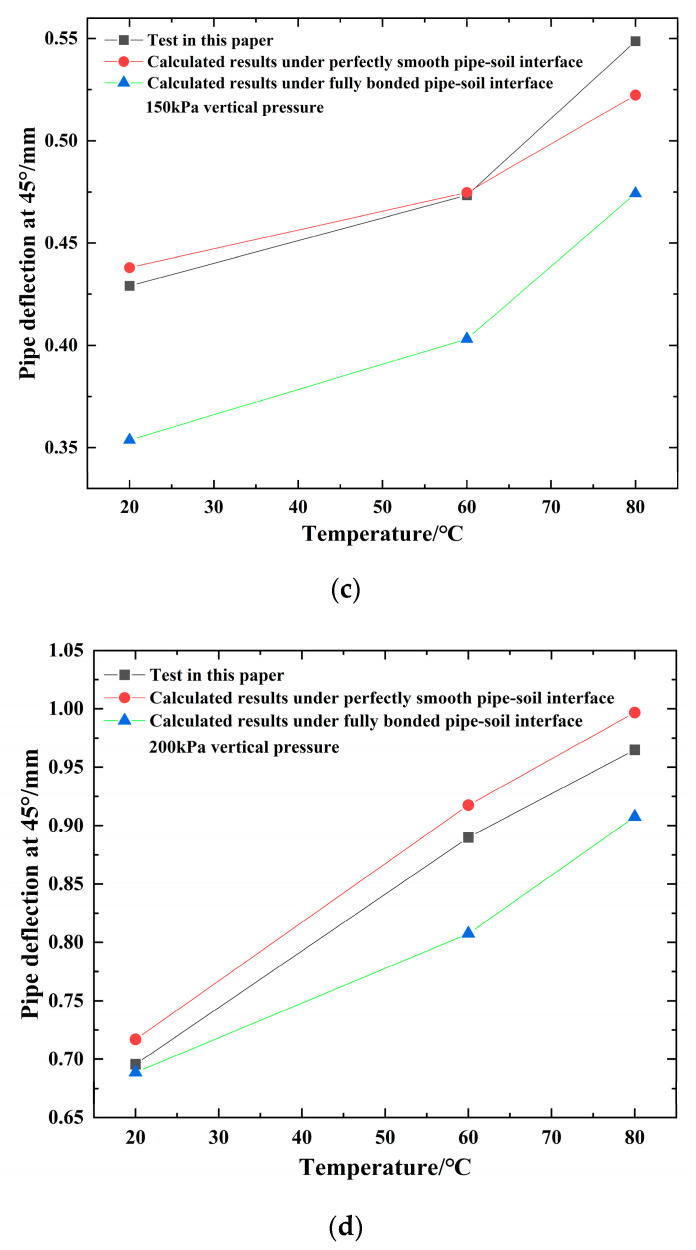
The variations of pipe deflections at 45° versus temperature: (**a**) 50 kPa; (**b**) 100 kPa; (**c**) 150 kPa; (**d**) 200 kPa.

The deformations of the pipe that were calculated are depicted in Figure 6 alongside the deflections that were measured. The variations in the inner diameter of the pipes at 45° were obtained by a laser displacement transducer placed inside the pipes. In most cases, there was a very high level of agreement in the vertical diameter change. As seen in Figure 6, several of outcomes of the experiments are more accurate than the results of the calculations. Because the effect of time was not taken into consideration during the calculation procedure, the experimental results provide the monitored quantity during a period of temperature control, whereas the effect of time was not taken into consideration during the experimental procedure. In addition, as shown in Figure 6, the findings of the experiment were quite similar to the results of the calculations for a completely smooth surface. This was because the sand that was used in the experiment was fairly smooth river sand, which is what was utilized in the experiment. Even though the pipe-soil interface was reasonably smooth, the friction on the pipe-soil interface increased when the vertical pressure increased because the soil surrounding the pipe was compressed. This is because the pipe was in a vertically inclined position. As a consequence, the findings of the experiment were closer to the results of the computational analyses when the interface was entirely bonded.

### 4.2. Comparison of the Buried HDPE Pipe Deflections in the Different Backfill Materials

Underground conduits are used for a variety of purposes, including culverts, oil lines, coal slurry lines, subway tunnels, water mains, gas lines, telephone and electrical conduits, sewer lines, drain lines, water mains, gas lines, heat distribution lines, and telephone and electrical conduits [32,36,37,38,39]. The properties of the soil envelope that surrounds a buried pipe system have a significant impact on the design of the buried pipe system. Throughout the course of history, soil was frequently employed in construction work. It is utilized in the construction of highways, embankments, and dams, among other things. Soil is an essential component in the construction of subterranean conduits such as sewers, culverts, tunnels, and other similar underground passageways because it serves not only as the material upon which the structure sits, but also as a substance that supports and transfers loads. The surface and gravitational loads are transferred to, from, and around the structure by the soil that surrounds it. The interplay between soil structure and soil mechanics has been the subject of a great deal of research and writing. It is usual practice to take into account factors such as the kind of soil, the density of the soil, and the depth of the installation [32].

There is a wide range of physical and chemical compositions among soils. In the tests of this paper, poorly-graded sand was used, as shown in Table 2. In landfills, buried pipes are typically installed using poorly-graded gravel, poorly-graded sand, and clay gravel [33,40].

The interface of the pipe and soil should lie between the two extreme conditions of perfectly smooth and fully bonded. Considering the two extreme conditions, the deflections of buried HDPE pipes can be predicted within a range by Zhang et al. [35]. Referring to Equation (2) in Zhang et al. [35], the upper and lower bounds of the deflection of the HDPE pipe in different sand and rocks can be determined under a perfectly-smooth pipe-soil interface and a fully-bonded pipe-soil interface, respectively.

The following parameters are used in the calculation: The outer and inner radii of the HDPE pipe are *R* = 0.055 m and *R*_0_ = 0.053 m, respectively; the elastic modulus and Poisson’s ratio of the soil are referred to [40]; the elastic modulus and Poisson’s ratio of the HDPE pipe are *E_c_* = 875 MPa and *v_c_* = 0.46, respectively. Based on Equation (2), the deflection of the HDPE pipe in different sand and rocks can be determined under a perfectly-smooth pipe-soil interface and a fully-bonded pipe-soil interface, respectively. The calculated results determined under 280 kPa vertical pressure are shown in Table 5, and the calculated results determined under 420 kPa vertical pressure are shown in Table 6.

As shown in Table 5 and Table 6, the maximum deflection of the HDPE pipe occurred at the vertical deflection (ΔD_v_) of the inner wall, considering both a perfectly-smooth pipe-soil interface and a fully-bonded pipe-soil interface. Comparing the four types of backfill materials, it is found that maximum deflection of the HDPE pipes is the greatest in clay gravel, followed by poorly-graded sand (used in the tests of this paper) and poorly-graded gravel.

## 5. Conclusions

HDPE pipes, which are essential components of landfill leachate collection and drainage systems, can sustain damage when subjected to significant amounts of earth pressure and elevated temperatures during construction and operation phases. With each additional layer of waste added to a landfill, there is a corresponding increase in the amount of pressure exerted on the HDPE pipes used for leachate collection. After completion of the trash filling process, heat generation occurs in landfills as a result of the breakdown of organic components. This heat generation adds to an increase in the temperature of the waste. For this reason, the strain and deformation need to be re-evaluated under conditions in which vertical pressure is applied and temperature increases. The following are the important inferences from this study:

(1) The wall thickness and profile of a pipe contribute uniquely to the overall stiffness of the pipe. Despite this, a broad classification of pipe behavior in relation to the stiffness of the soil in the surrounding area is beneficial.
(2) The strain distributions around a pipe change from a V-shape to a U-shape as the temperature increases from 20 °C to 60 °C and from 60 °C to 80 °C. The deformation profile of the pipe changes from an ellipse to a rectangle when the strain distributions change from a V shape to a U shape.
(3) Even though the deformation profile of a buried HDPE pipe shifts from an ellipse to a rectangle as the ambient temperature rises and the location of the maximum circumferential strain shifts, the current analytical method that considers two extreme interfaces is able to capture the deformation in the model test well.
(4) The findings presented in this paper demonstrate that the elevated temperatures prevalent in landfills lead to deformations in HDPE pipes that result in nonelliptical pipe shapes. Therefore, while designing a pipe that collects leachate from a landfill, in addition to taking into consideration the maximum vertical and horizontal deformations, one should also take into consideration the stress and strain to determine whether the pipe is stable.

## Figures and Tables

**Table 1:** Material properties of the HDPE pipe tested in this paper.

Parameter	HDPE Pipe
Outer diameter	110 mm
Wall thickness	10 mm (SDR = 10) 4.2 mm (SDR = 26)
Density	0.955 g/cm^3^
Melting flow rate (190 °C, 5 kg)	0.46 g/10 min
Elongation at break	674%
Longitudinal reversion rate (100 °C, 2 h)	0.7%
Oxidative induction time (200 °C)	70 min

**Table 2:** Parameters of sand used in tests.

Parameter	Sand
Specific gravity	2.65
Maximum dry density (g/cm^3^)	1.77
Minimum dry density (g/cm^3^)	1.60
As-placed dry density (g/cm^3^)	1.75
Moisture content (%)	3.72
D_60_ (mm)	0.732
D_30_ (mm)	0.406
D_10_ (mm)	0.246
C_u_	2.976
C_c_	0.915
Backfill type	Poorly-graded sand (SP)

**Table 3:** List of tests and details.

No.	Backfill Materials	Labels	SDR of the HDPE Pipe	Maximum Pressure (kPa)	Temperature (°C)
1	Poorly graded medium-coarse sand	SP1	11	300	20
2		SP2	26	300	20
3		SP3		50	20, 60, 80
4		SP4		100	20, 60, 80
5		SP5		150	20, 60, 80
6		SP6		200	20, 60, 80

**Table 4:** The creep measurements of the HDPE pipe in the duration of the test.

Vertical Pressure	Pipe Deformation	20 °C	60 °C 3 Days	Increased Factor	80 °C 3 Days	Increased Factor
50 kPa	ΔD_v_	−0.1983 mm	−0.2357 mm	1.19	−0.2604 mm	1.31
	ΔD_h_	0.1482 mm	0.1750 mm	1.18	0.1865 mm	1.26
100 kPa	ΔD_v_	−0.4329 mm	−0.4774 mm	1.09	−0.5225 mm	1.21
	ΔD_h_	0.2972 mm	0.3211 mm	1.08	0.3418 mm	1.15
150 kPa	ΔD_v_	−0.5781 mm	−0.6345 mm	1.09	−0.7277 mm	1.26
	ΔD_h_	0.4417 mm	0.4785 mm	1.08	0.5414 mm	1.23
200 kPa	ΔD_v_	−0.8718 mm	−0.9677 mm	1.11	−1.1806 mm	1.22
	ΔD_h_	0.6749 mm	0.7559 mm	1.12	0.9297 mm	1.23

**Table 5:** The deflection of the buried HDPE pipe in different backfill materials under 280 kPa vertical pressure.

Soil Type		Perfectly-Smooth Pipe-Soil Interface (Upper Bound of Deflection)		Fully-Bonded Pipe-Soil Interface (Lower Bound of Deflection)	
		Outer Wall Deflection/m	Inner Wall Deflection/m	Outer Wall Deflection/m	Inner Wall Deflection/m
Poorly-graded gravel	ΔD_h_	0.000899	0.000881	0.000575	0.000549
	ΔD_v_	−0.001096	−0.001113	−0.000771	−0.000780
Poorly-graded sand (Used in the tests of this paper)	ΔD_h_	0.001210	0.001191	0.000936	0.000913
	ΔD_v_	−0.001422	−0.001441	−0.001148	−0.001162
Clay gravel	ΔD_h_	0.001617	0.001597	0.001233	0.001209
	ΔD_v_	−0.001828	−0.001847	−0.001440	−0.001460

**Table 6:** The deflection of the buried HDPE pipe in different backfill materials under 420kPa vertical pressure.

Soil Type		Perfectly-Smooth Pipe-Soil Interface (Upper Bound of Deflection)		Fully-Bonded Pipe-Soil Interface (Lower Bound of Deflection)	
		Outer Wall Deflection/m	Inner Wall Deflection/m	Outer Wall Deflection/m	Inner Wall Deflection/m
Poorly-graded gravel	ΔD_h_	0.001011	0.000984	0.000658	0.000620
	ΔD_v_	−0.001305	−0.001330	−0.000952	−0.000966
Poorly-graded sand (Used in the tests of this paper)	ΔD_h_	0.001315	0.001287	0.001035	0.001002
	ΔD_v_	−0.001631	−0.001659	−0.001351	−0.001374
Clay gravel	ΔD_h_	0.001572	0.001543	0.001235	0.001201
	ΔD_v_	−0.001888	−0.001916	−0.001552	−0.001573

## Data Availability

Not applicable.
